# Spatial/Temporal Variations and Source Apportionment of VOCs Monitored at Community Scale in an Urban Area

**DOI:** 10.1371/journal.pone.0095734

**Published:** 2014-04-22

**Authors:** Chang Ho Yu, Xianlei Zhu, Zhi-hua Fan

**Affiliations:** 1 Division of Exposure Science, Environmental and Occupational Health Sciences Institute, Rutgers University, Piscataway, New Jersey, United States of America; 2 College of Geosciences, China University of Petroleum, Beijing, People’s Republic of China; Tsinghua University, China

## Abstract

This study aimed to characterize spatial/temporal variations of ambient volatile organic compounds (VOCs) using a community-scale monitoring approach and identify the main sources of concern in Paterson, NJ, an urban area with mixed sources of VOCs. VOC samples were simultaneously collected from three local source-dominated (i.e., commercial, industrial, and mobile) sites in Paterson and one background site in Chester, NJ (located ∼58 km southwest of Paterson). Samples were collected using the EPA TO-15 method from midnight to midnight, one in every sixth day over one year. Among the 60 analyzed VOCs, ten VOCs (acetylene, benzene, dichloromethane, ethylbenzene, methyl ethyl ketone, styrene, toluene, m,p-xylene, o-xylene, and p-dichlorobenzene) were selected to examine their spatial/temporal variations. All of the 10 VOCs in Paterson were significantly higher than the background site (p<0.01). Ethylbenzene, m,p-xylene, o-xylene, and p-dichlorobenzene measured at the commercial site were significantly higher than the industrial/mobile sites (p<0.01). Seven VOCs (acetylene, benzene, dichloromethane, methyl ethyl ketone, styrene, toluene, and p-dichlorobenzene) were significantly different by season (p<0.05), that is, higher in cold seasons than in warm seasons. In addition, dichloromethane, methyl ethyl ketone, and toluene were significantly higher on weekdays than weekend days (p<0.05). These results are consistent with literature data, indicating the impact of anthropogenic VOC sources on air pollution in Paterson. Positive Matrix Factorization (PMF) analysis was applied for 24-hour integrated VOC measurements in Paterson over one year and identified six contributing factors, including motor vehicle exhausts (20%), solvents uses (19%), industrial emissions (16%), mobile+stationery sources (12%), small shop emissions (11%), and others (22%). Additional locational analysis confirmed the identified sources were well matched with point sources located upwind in Paterson. The study demonstrated the community-scale monitoring approach can capture spatial variation of VOCs in an urban community with mixed VOC sources. It also provided robust data to identify major sources of concern in the community.

## Introduction

Volatile organic compounds (VOCs) are a group of air pollutants emitted from multiple types of anthropogenic sources, such as refineries, chemical factories, gas stations, dry cleaners, paint shops and diesel/gasoline-powered vehicles as well as biogenic sources. Previous studies have suggested associations between some VOCs in ambient air and adverse health outcomes, such as asthma [1], [2], [3]. As reported by many studies, “hot spots” of VOCs may exist due to presence of various local emission sources in urban communities [4], [5], [6], [7], [8], [9], [10], [11]. However, VOCs data measured at community levels are limited. Thus, to better understand community exposures to ambient VOCs and associated health effects, monitoring of VOCs at community scale and characterization of their spatial/temporal variations are needed.

Many urban areas have mixed emission sources of VOCs, including mobile, commercial and industrial sources. However, gross industrial VOC emissions rather than speciated VOC emissions are usually reported to local air pollution control agencies [12]. Moreover, emission data are often obtained from estimation rather than true measurements, and many are not even available for small facilities. Therefore, the lack of detailed emission data prevents the evaluation of the impact of any emission sources on local VOC air pollution, and thus limits the development of effective controlling strategies. Furthermore, previous VOC source apportionment studies were extensively conducted using the measurements collected in the summer (e.g., Photochemical Assessment Monitoring Stations (PAMS)) [13], [14], [15]. Therefore, the results obtained from those studies primarily represented the sources of VOCs in the summer, not for other seasons. Given such, measurement of VOCs at community scale throughout one year and apportionment of their sources are needed.

This study aimed to characterize spatial and temporal variations of air toxics at community-scale in an urban area, i.e. Paterson, NJ, with mixed sources of VOCs. The emission sources included industrial, commercial, mobile and residential sources [12], [16], [17]. Among the monitored 60 VOCs, ten VOCs (acetylene, benzene, dichloromethane, ethylbenzene, methyl ethyl ketone, styrene, toluene, m,p-xylene, o-xylene and p-dichlorobenzene) that were detected over 75%, had toxicities and/or known sources in the study area were specifically selected for examining spatial and temporal variations. Also, the contributions from different VOC sources to local air pollution were estimated using Positive Matrix Factorization (PMF) analysis. Our study demonstrated that the community-scale monitoring approach could effectively capture local-dominated VOC sources in urban communities with mixed emission sources. In addition, to our best knowledge, this is the first attempt to conduct VOC source apportionment using measurements collected over a course of one year. Therefore, the major sources identified in our study reflected seasonal changes in the study area, and our approach provided more accurate estimate of the contribution of local VOC emission sources to community air pollution when compared to those obtained from the summer measurements only. Therefore, our study approach is more helpful for the development of effective strategies to control and reduce community air pollution.

## Methods

### Study Area

Paterson is located in Passaic county of NJ, with high population density (6,826/km^2^ with a total population of 146,199 in US Census of 2010) and socio-economically disadvantaged populations [18]. It is composed of sections that are dominated by industrial (e.g., textiles, dyes, chemicals, metal fabrication/refinishing/recovery; plastics, printing, electronics, paper/food products, etc.), commercial (e.g., dry cleaners, fast food restaurants, photo labs, commercial heating/boilers, nail salons, print shops, etc.) and mobile sources (e.g., US I-80, Route 19 and County Route 649, 639 and 648).

### Monitoring Sites

Three monitoring sites, i.e., commercial, industrial and mobile sampling sites, were selected for sampling based on Geographic Information System (GIS) layers of population density, road type, source proximity, traffic count and land use type, as well as accessibility, security of the sampling systems and availability of electricity. The site map can be found in our previous publication [17]. Briefly, the industrial site was located at a public school in northern Paterson, about 0.1–1.0 km south-southeast of a highly industrialized area known as Bunker Hill. This area hosts a variety of industrial facilities, emitting toluene, methyl ethyl ketone (MEK), methyl isobutyl ketone (MIBK), xylenes, ethylbenzene, and general VOCs. The monitoring site for the mobile source-dominated area was located at a public school in southwestern Paterson. Several major roadways are located within 0.8 km of the school, including the US Interstate Route 80 & 19, a major NJ Transit Bus Depot and an active rail yard/line. The commercial monitoring site was located at a health department building near the shopping district in downtown Paterson. There are many typical urban commercial sources, such as dry cleaners, fast food restaurants, photo finishing, commercial heating/boilers, nail salons, print shops, etc. Monitoring devices were placed on the rooftop (approximately 10∼13 m above the ground) of the school/building given space and security restrictions. The background site was located in an open field in Chester, NJ, about 58 km west/southwest of Paterson. This site is designated as the background/rural site for the Urban Air Toxic Monitoring Program (UATMP) by the New Jersey Department of Environmental Protection (NJDEP) and has been in operation since 2001.

This study was jointly conducted by Rutgers University and the NJDEP. The NJDEP obtained the approval from the Board of Education of Paterson Public School District to place air toxics monitoring equipment on the roof of two school buildings, to capture mobile and industrial source-oriented emissions. The NJDEP also obtained approval from the Paterson Public Health Department to place the air monitors at the local building in downtown of Paterson. The NJDEP allowed the state-designated background site in Chester for air sampling. Due to confidentiality concerns, specific location information (e.g., GPS coordinates) is not provided in this manuscript.

### Sample Collection and Measurement

One year field sampling was conducted from November 18, 2005 to December 19, 2006. The sampling frequency was one in six days, and the sampling duration was 24 hours, from midnight to midnight. The study was designed to represent community’s exposure to air toxics in an urban community; thus, one full day monitoring (i.e., 24-hour sampling) was employed. The study employed the same UATMP sampling frequency and schedule, which aims to capture trends of air toxic pollutants, so that we could compare the data collected from this study to other UATMP urban sites (e.g., Camden, Elizabeth and New Brunswick) in NJ. Ambient VOCs were collected using a stainless steel canister with an air sampler (ATEC model 2200, Malibu, CA), following the EPA TO-15 method [19]. After sample collection, the canister was sent to Environmental Research Group (ERG, Morrisville, NC) for analysis. The delivered samples were analysed using gas chromatography-mass spectrometry (GC-MS) within 3 days. Besides the 10 target compounds, other 50 species were analysed by ERG. All sampling and analysis procedures, including canister cleaning, calibration of analytical system and quantification of target compounds, were exclusively conducted by ERG, an US EPA national contract laboratory. All quality assurance/quality control (QA/QC) procedures have been overseen and documented by the USEPA.

### QA/QC

Twenty five duplicate samples (∼10% out of the 209 regular samples) were collected side-by-side during the study period, and the measurement precision for each VOC species was evaluated by the absolute percent difference (%Diff) between the two co-located samples. The difference was calculated using the following equation (1):
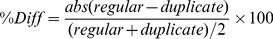
(1)


Good precision was obtained for most VOCs, except acrolein. The %Diff was less than 20% for the majority of the target compounds. The precision of acrolein, however, was poor, with %Diff of 55%. It was suspected that the poor precision of acrolein may be partially contributed by artificial formation of acrolein in the canister during storage [20].

The method detection limit (MDL) was calculated as the product of the standard deviation (SD) of seven replicate analyses and the Student’s t-test value for 99% [19]. The MDLs are reported in Table 1. Prior to field sampling, all canisters were cleaned at the analytical laboratory and delivered to sampling sites vacuumed. Therefore, field blank sample collection was not applicable for this type of canister method. Thus, field blanks were not collected and blank subtraction was not performed for the data reported in this study.

**Table 1:** Ambient VOCs concentrations (µg/m^3^) measured at the three monitoring sites in Paterson and one background site in Chester.

VOC Compounds	N	Avg	SD	Min	Med	Max	MDL	>MDL(%)	ND(%)
1,1,1,-Trichloroethane^a^	209	0.15	0.09	0.05	0.11	0.98	0.02	99	0
1,2,4-Trimethylbenzene^a^	209	0.54	0.59	0.01	0.39	4.38	0.02	89	9
1,3,5-Trimethylbenzene^a^	209	0.17	0.17	0.01	0.15	1.23	0.02	85	12
1,3-Butadiene^a^	209	0.15	0.18	0.01	0.11	1.35	0.01	84	14
Acetonitrile^a^	209	0.53	0.83	0.08	0.20	6.54	0.17	52	44
Acetylene^a^	209	1.15	1.24	0.02	0.77	8.22	0.03	100	0
Acrolein^a^	209	0.79	0.74	0.11	0.62	3.86	0.25	76	20
Benezene^a^	209	1.13	0.89	0.22	0.90	6.52	0.02	100	0
Carbon Disulfide	209	0.72	1.46	0.01	0.09	16.0	0.03	73	27
Carbon Tetrachloride^a^	209	0.61	0.18	0.06	0.57	1.07	0.06	100	0
Chloroethane	209	0.04	0.06	0.01	0.03	0.58	0.02	59	34
Chloroform^a^	209	0.19	0.21	0.01	0.15	1.52	0.02	75	22
Chloromethane^a^	209	1.15	0.19	0.56	1.16	1.78	0.03	100	0
Dichlorodifluoromethane^a^	209	2.89	0.64	1.19	2.83	7.59	0.03	100	0
Dichloromethane^a^	209	0.91	1.17	0.03	0.56	7.18	0.06	98	1
Dichlorotetrafluoroethane	209	0.12	0.03	0.07	0.14	0.14	0.02	100	0
Ethylbenzene^a^	209	0.57	0.84	0.04	0.35	9.04	0.02	100	0
Methyl Ethyl Ketone^a^	209	1.88	2.07	0.07	1.18	14.0	0.13	91	8
Methyl Isobutyl Ketone^a^	209	0.37	0.48	0.01	0.25	3.57	0.03	78	21
Methyl tert-Butyl Ether^a^	209	0.45	1.09	<0.01	0.14	7.93	0.01	68	32
Propylene^a^	209	1.05	0.96	0.12	0.86	6.90	0.02	100	0
Styrene^a^	209	0.16	0.16	0.02	0.13	0.98	0.04	77	13
Tetrachloroethylene^a^	209	0.48	0.56	0.04	0.34	5.10	0.08	82	7
Toluene^a^	209	5.27	5.83	0.19	3.62	32.4	0.02	100	0
Trichloroethylene^a^	209	0.10	0.14	0.03	0.03	1.46	0.05	36	54
Trichlorofluoromethane^a^	209	2.02	1.19	0.68	1.69	11.2	0.04	100	0
Trichlorotrifluoroethane^a^	209	0.79	0.17	0.38	0.77	1.61	0.09	100	0
m,p-Xylene^a^	209	1.77	3.67	0.04	0.91	40.9	0.04	100	0
n-Octane^a^	209	0.20	0.20	0.01	0.14	1.50	0.03	85	13
o-Xylene^a^	209	0.56	0.72	0.01	0.39	6.65	0.02	97	1
p-Dichlorobenzene^a^^,^ b	195	0.24	0.23	0.02	0.18	1.39	0.04	75	23

a These VOCs were selected for the PMF analysis based on S/N ratio >2 and detection >50% in pooled Paterson data.

b High concentrations (N = 14) monitored at the commercial site in the period of 9/26/2006**∼**12/17/2006 were excluded.

### Data Analyses

#### Data selection and substitution

The number of samples (i.e., 209) presented in Table 1 included all of the samples collected from the three monitoring sites in Paterson and the one background site in Chester. Among the 60 VOCs analyzed by the EPA TO-15 method, ten VOCs (acetylene, benzene, dichloromethane, ethylbenzene, MEK, styrene, toluene, m,p-xylene, o-xylene and p-dichlorobenzene), which were detected over 75% during the course of monitoring period, toxic and/or having known sources in the study area, were specifically selected for analyzing their spatial/temporal variations. In addition to the 10 selected VOCs, twenty one VOCs that were detected over 50% or used for the PMF analysis were also reported in Table 1. For the non-detects (ND), we replaced them with a half of the MDL in data analysis. Since, for the 10 target species, more than 75% of the samples were detected above MDL, the substitution of ND with a half of MDL was not expected to significantly affect the spatial/temporal variations for these target species.

The data were not normally distributed; thus, non-parametric approaches were used for data analysis. Specific descriptions of each analysis are presented below.

#### Spatial and temporal variability

Descriptive statistics, including mean, standard deviation, minimum, median and maximum, were performed to characterize the distributions of the VOC concentrations. To examine site and seasonal differences, non-parametric Kruskal-Wallis test (two-sided) was conducted. If the difference was found to be significant (p<0.05), pairwise multiple comparison tests were followed with the significance determined by Bonferroni’s corrected alpha (i.e., 0.05/6 = 0.0083). For the difference between weekday and weekend, Wilcoxon Rank-sum test (two-sided) was conducted. To increase statistical power, temporal variability (i.e., seasonal differences and weekday and weekend differences) was conducted on pooled data from the three sites in Paterson. All statistical analyses were conducted by SAS (v. 9.2).

#### Positive Matrix Factorization (PMF) model

As the study measured VOC concentrations in Paterson over one year period, source-receptor relationships were explored using a mass balance approach to identify and to apportion the sources of ambient VOC concentrations in Paterson, with a consideration of seasonal variation in emission sources. A PMF model (v. 3.0) was used to provide source profiles and contributions to the measured data. Detailed explanations and equations used in the PMF analysis can be found elsewhere [21], [22]. The predicted mass fractions and source factors obtained from the PMF analysis can be used to identify major sources that significantly contribute to the VOC air pollution in Paterson.

Prior to performing PMF analysis with pooled Paterson data, spatial correlations were examined first to check the pooled VOC data might be correlated each other spatially. Moran’s I and Geary’s C tests were conducted for the VOC data sets, and the results indicated that the spatial autocorrelations were not significant (p>0.05). Therefore, daily arithmetic mean for each VOC species was calculated by averaging the measurements from the three monitoring sites in Paterson. This allowed to estimate VOC source contributions in Paterson over entire study period (i.e., ∼1 year). The uncertainty of each species was calculated using MDLs and error fraction, assuming 20% for all species in this study [23]. Among the 60 species measured in the study, twenty eight compounds that were detected more than 50% and had a signal-to-noise (S/N) ratio greater than 2 [22], [24] were selected for PMF analysis (Table 1). Any non-detects in the input data were substituted with a half of the MDL. There was no missing sampling date for the PMF input database. In addition, the total VOC concentration, summing the 28 selected VOCs, were calculated and included as an independent variable in the PMF model to provide direct mass apportionments [13], [14].

The PMF analysis suggested six factor profiles as appropriate source types in the source-receptor relationships. Different number of source profiles (e.g., five and seven factors) was additionally conducted during the analysis to identify proper number of sources in the given data sets [25]. In the seven-source model, the final model produced a negative constant, indicating too many sources were used. In the five-source model, the mobile and industrial emissions were not further separated. Therefore, the six-source model provided the most physically reasonable source profiles. As a part of finalizing best-fit source profiles, we utilized emission inventory (EI) data in Paterson as well as source identification results published previously for particulate matter less than 10 µm in diameter (PM_10_) [17] and polycyclic aromatic hydrocarbons (PAHs) [16] that were concurrently measured in the same study. The rotational ambiguity was further investigated by inspecting pairs of the final factors in different FPEAK value range to avoid subjective bias in some extent. Sensitivity analyses, such as running a PMF with the 5-factor and 7-factor models as well as 5% extra modeling uncertainty, were conducted to verify whether the selected 6-factors were robust in the final form. The bootstrap running for the selected 6-factor model was repeated in 100 times to check if the factors were stable and consistent. Also, the positive FPEAK values (0.1∼0.5) were used to sharpen the ambiguous source profiles in the base run model. After these additional tests, the final 6 factors remained constant. In this way, subjective bias was reduced significantly [26], [27].

To help identifying the likely locations of the PMF-identified sources, a conditional probability function (CPF) was calculated. This approach was previously conducted by Kim et al. [28]. Briefly, daily fractional mass contribution from each source was used to minimize the effect of atmospheric dilution, rather than the absolute source contribution from all sources. The same daily fractional contribution was assigned to each hour in a given day to match the hourly wind data. Specifically, the CPF was defined as the following equation (2):
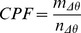
(2)where m_Δθ_ is the number of occurrence from wind sector Δθ, and n_Δθ_ is the total number of data from the same wind direction. In this study, the highest 10 percentile of the daily fractional contribution from each source was chosen. Corresponding hourly wind data, except calm winds (<1 m/sec), were counted by the sector of 10 degrees. A CPF value close to 1.0 for a given sector indicates a high probability of a source located in that direction.

## Results and Discussion

### Spatial and Temporal Variability

#### Spatial variability

**Figure 1:**
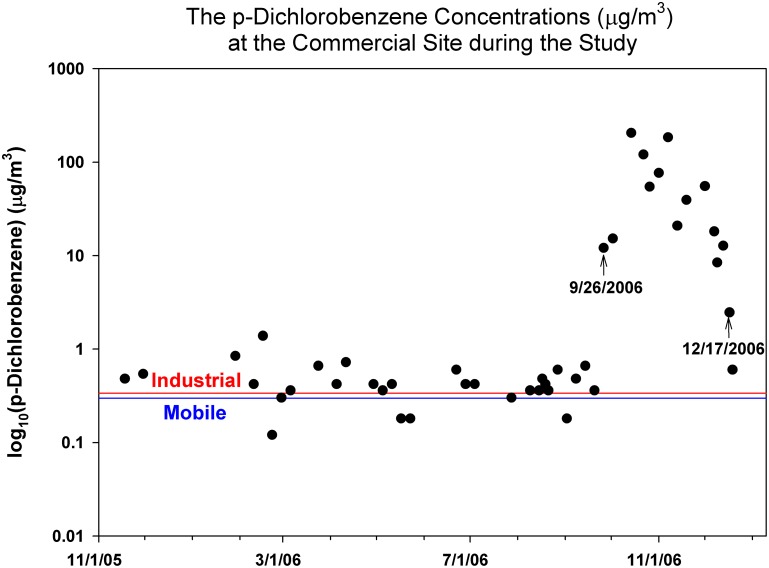
The monitored p-dichlorobenzene concentrations at the commercial site during the study period (11/18/2005∼12/19/2006). The averaged p-dichlorobenzene concentrations at industrial (dashed line) and mobile (dotted line) sites for the monitoring period were added for references.

For p-dichlorobenzene, there was a striking difference among commercial (AVG±SD [Min–Max]: 18.7±45.2 [0.12–205] µg/m^3^), industrial (0.34±0.20 [0.04–1.02] µg/m^3^) and mobile (0.30±0.17 [0.06–0.78] µg/m^3^) sites. The mean concentration at the commercial site was two orders of magnitude higher than the other two sites. The high concentrations at the commercial site were driven by the high concentrations (N = 14; 59.0±66.1 [2.47–205] µg/m^3^) observed between September 26 and December 17, 2006 (Figure 1). Because of the measured high p-dichlorobenzene concentrations at the commercial site, additional VOC monitoring was conducted from April 2010 to May 2011 at this site. The study monitored VOC concentrations every six days over a course of one year at the commercial site, and five spatial saturation sampling (SSS) campaigns around the commercial site were carried out over the one year monitoring period. For the SSS sampling, organic vapor monitor (OVM, 3M, St. Paul MN) passive badge were deployed for three days in 23 locations within the city in a grid-like fashion around the commercial monitoring site. There was no spike p-dichlorobenzene concentrations measured in any sampling campaign (N = 37; 0.29±0.23 [0.03–1.28] µg/m^3^), and the concentrations obtained from the SSS campaign were similar to other urban areas in NJ (i.e., Camden, Elizabeth, and New Brunswick). The study concluded that the high p-dichlorobenzene concentrations were one time event, and indoor sources, such as room deodorizer or moth repellent from the building, might result in those high measurements. Thus, the measurements from this particular period were excluded for the spatial/temporal variations analysis and PMF analysis.

The concentrations of the 10 selected VOC species at each sampling site and the comparison results among the 4 sampling sites are presented in Table 2. All of the 10 VOCs showed significant differences among the four sampling sites (p<0.05), particularly, the concentrations measured in Paterson were much higher than the background site in Chester. The VOC concentrations in Paterson were similar to those in other urban communities, i.e., Camden, Elizabeth and New Brunswick, across NJ [12]. Multiple comparison tests (i.e., Kruskal-Wallis test) confirmed that the spatial variability was resulted from significant differences between the background site and the three monitoring sites in Paterson. The difference between two geographical locations (i.e., higher concentrations in Paterson than in Chester) indicated the impact of local sources of VOC in Paterson, consistent with the VOC source information in Paterson documented by the NJDEP. As described in the Introduction, the NJDEP has identified many industrial sources of these species in Paterson, such as emissions of toluene (10.5 tons/year), MEK (0.1 tons/year), xylenes (8.4 tons/year), ethylbenzene (1.6 tons/year), styrene (0.5 tons/year) and benzene (0.2 tons/year) from industrial facilities. In contrast, there are no identified industrial sources of air pollution near the Chester site [12].

**Table 2:** Descriptive statistics and spatial differences for the 10 VOCs (µg/m^3^) monitored at the four local-source dominated sites in the study.

	N	Mean	SD	Min	Med	Max	p-value^a^	MultipleComparison^b^		N	Mean	SD	Min	Med	Max	p-value^a^	MultipleComparison^b^
**Acetylene**									**Styrene**								
Background	69	0.52	0.35	0.02	0.43	1.76	<.0001	A	Background	69	0.07	0.06	0.02	0.04	0.34	<.0001	A
Commercial	45	1.76	1.72	0.34	1.16	8.22		B	Commercial	45	0.28	0.24	0.02	0.21	0.98		B
Industrial	45	1.20	1.10	0.20	0.97	6.89		B	Industrial	45	0.16	0.11	0.02	0.13	0.60		C
Mobile	50	1.41	1.27	0.27	1.11	6.72		B	Mobile	50	0.17	0.11	0.02	0.13	0.60		B C
**Benzene**									**Toluene**								
Background	69	0.52	0.22	0.22	0.48	1.19	<.0001	A	Background	69	0.71	0.54	0.19	0.53	3.17	<.0001	A
Commercial	45	1.81	1.15	0.54	1.44	6.52		B	Commercial	45	7.98	6.06	1.06	6.37	32.4		B
Industrial	45	1.11	0.68	0.35	0.90	4.16		C	Industrial	45	6.46	4.94	0.45	5.47	25.6		B
Mobile	50	1.36	0.82	0.35	1.21	4.99		B C	Mobile	50	8.06	6.60	0.87	6.49	31.4		B
**DCM**									**m,p-Xylene**								
Background	69	0.33	0.25	0.03	0.28	1.60	<.0001	A	Background	69	0.30	0.21	0.04	0.26	1.13	<.0001	A
Commercial	45	1.15	1.26	0.17	0.80	7.18		B	Commercial	45	4.62	6.99	0.43	2.39	40.9		B
Industrial	45	1.31	1.46	0.17	0.70	6.34		B	Industrial	45	1.51	1.28	0.17	1.35	7.17		C
Mobile	50	1.14	1.28	0.24	0.71	6.83		B	Mobile	50	1.45	1.09	0.26	1.24	6.82		C
**EB**									**o-Xylene**								
Background	69	0.14	0.08	0.04	0.13	0.43	<.0001	A	Background	69	0.13	0.08	0.01	0.13	0.43	<.0001	A
Commercial	45	1.28	1.50	0.17	0.83	9.04		B	Commercial	45	1.20	1.19	0.17	0.87	6.65		B
Industrial	45	0.53	0.38	0.09	0.48	2.22		C	Industrial	45	0.55	0.42	0.09	0.48	2.48		C
Mobile	50	0.54	0.34	0.13	0.48	2.17		C	Mobile	50	0.57	0.40	0.13	0.48	2.56		C
**MEK**									**p-DCB**								
Background	69	1.08	1.22	0.07	0.91	8.09	<.0001	A	Background	69	0.04	0.04	0.02	0.02	0.24	<.0001	A
Commercial	45	2.76	2.11	0.07	2.27	10.0		B	Commercial	31	0.47	0.24	0.12	0.42	1.39		B
Industrial	45	2.77	3.06	0.07	1.77	14.0		B	Industrial	45	0.34	0.20	0.02	0.30	1.02		C
Mobile	50	1.41	1.09	0.07	1.03	4.72		A	Mobile	50	0.30	0.17	0.06	0.27	0.78		C

a Differences within the four sampling sites were conducted using the Kruskal-Wallis test and pairwise multiple comparison tests (Wilcoxon rank-sum test) were followed, if the difference was significant (p<0.05).

b Different letters mean significant differences (p<0.0083) among the four monitoring sites.

Abbreviation in the table: DCM: Dichloromethane, EB: Ethylbenzene, MEK: Methyl Ethyl Ketone, p-DCB: p-Dichlorobenzene.

Among the three sites in Paterson, ambient ethylbenzene, m,p-xylene, o-xylene and p-dichlorobenzene concentrations measured at the commercial site were significantly higher than at the industrial and mobiles sites (p<0.01). Specifically, benzene and styrene concentrations were significantly higher at the commercial site than at the industrial site (p<0.01) and marginally higher than at the mobile site (p = 0.04 and 0.01, respectively). These results indicated additional sources of these species at the commercial site. According to the NJDEP’s EI database, industrial facilities in Paterson reported significant emissions of xylenes, ethylbenzene and styrene to ambient air. Acetylene, benzene, ethylbenzene, toluene, m,p-xylene and o-xylene can be emitted from gasoline-powered vehicles [29]. The commercial site was located in downtown, Paterson, close to busy local roads with high volume of traffic. No significant spatial differences were found for acetylene, dichloromethane, MEK and toluene in Paterson.

#### Seasonal variability

**Figure 2:**
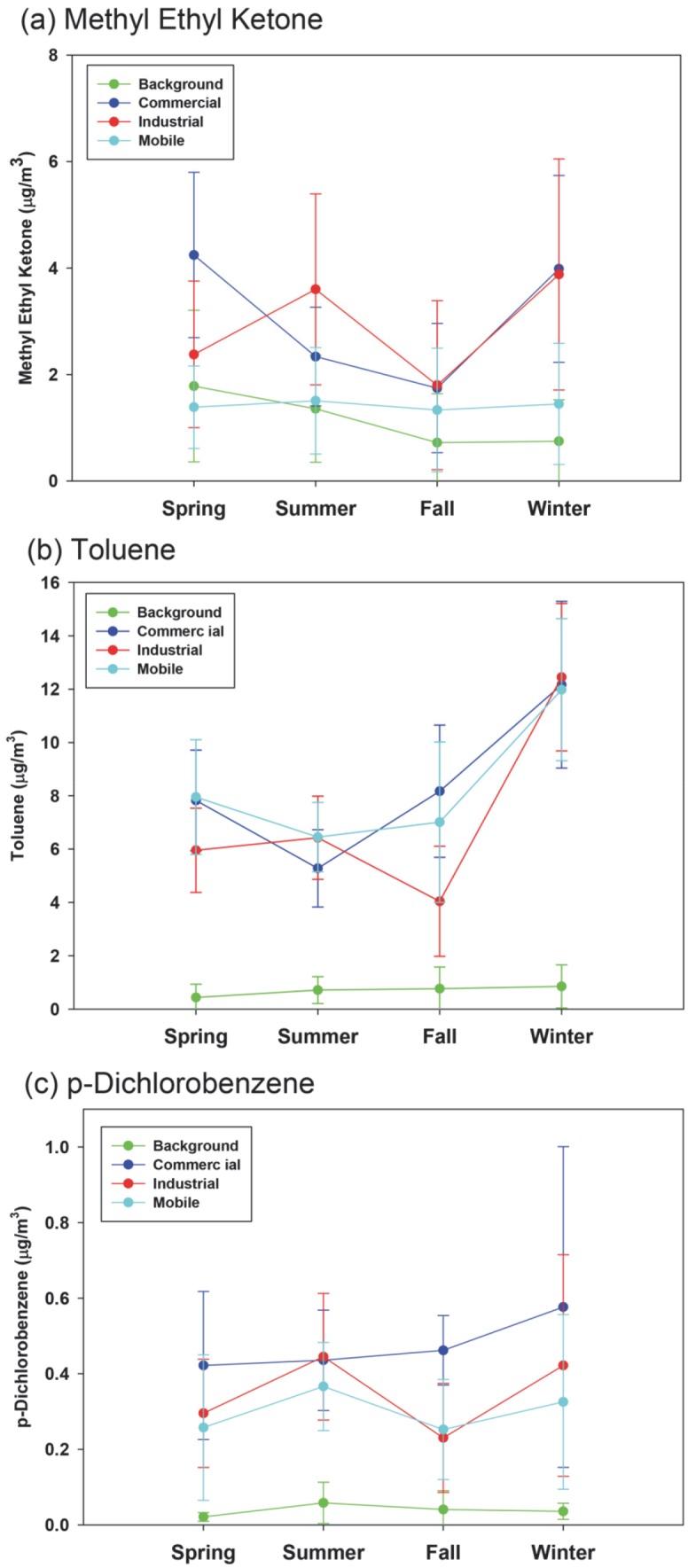
Seasonal variation (mean±SE [standard error]) of (a) MEK, (b) toluene, and (c) p-dichlorobenzene at each sampling site.

The seasonal differences were examined on the selected 10 VOCs. We found significant seasonal differences (p<0.05) for acetylene, benzene, dichloromethane, MEK, styrene, toluene and p-dichlorobenzene, and MEK, toluene and p-dichlorobenzene were selected for illustration (Figure 2). However, specific seasonal patterns were different by species. The winter concentrations of acetylene, benzene and toluene were higher than in other seasons; meanwhile, the summer p-dichlorobenzene concentrations were higher than in other seasons. Benzene is emitted from numerous industrial operations and mobile sources [30] and heating [31], which may explain the seasonal variation, i.e., higher emissions of benzene from combustion sources under low temperature in cold seasons. The higher winter concentrations were most likely due to winter heating, lower photochemical degradation and meteorological conditions (i.e., inversions and low mixing heights) in cold seasons [32], [33], [34]. The spring concentrations of dichloromethane, ethylbenzene, styrene, m,p-xylene and o-xylene were lower than in other seasons. On the other hand, MEK concentrations were the lowest in the fall and higher in warm seasons than in cold seasons. The winter MEK concentrations were also found to be high in the industrial and commercial areas. Main sources of MEK in ambient air are industrial sources (MEK is a common solvent); thus higher concentrations of MEK in warm seasons are expected due to evaporation. The high concentration in the winter is probably due to low photochemical decay rate and low mixing height by inversion, as stated above. In addition, the “large” increase of MEK (AVG±SD, 1.78±2.03 µg/m^3^) in the spring at the background site was driven by one high value (8.09 µg/m^3^). If this suspected outlier (Grubbs’ test for outliers, p<0.01) was removed, the background MEK concentrations in the spring (1.30±0.95 µg/m^3^) were similar to those at the mobile site (1.38±0.60 µg/m^3^).

#### Weekday vs. weekend difference

**Figure 3:**
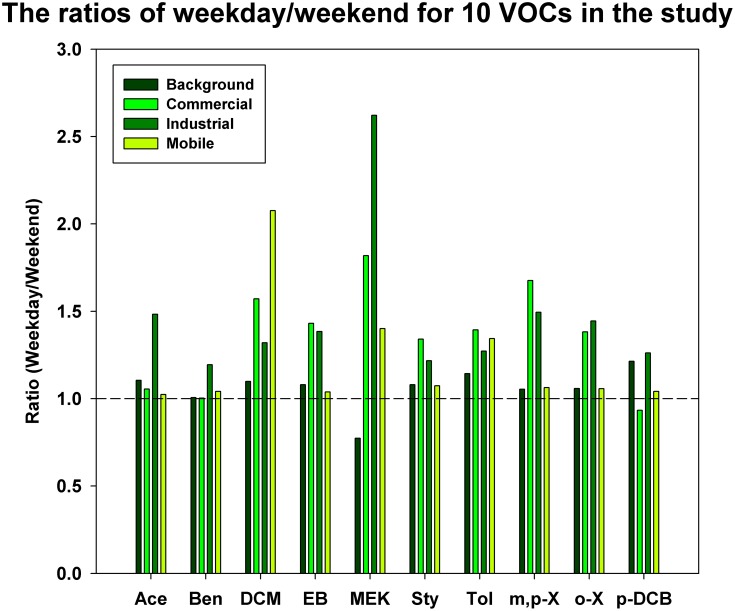
The ratios of weekday/weekend mean concentrations by each sampling site. The dotted line indicates the equivalent concentration for weekday and weekend measurements. Abbreviation in the figure: Ace: Acetylene, Ben: Benzene, DCM: Dichloromethane, EB: Ethylbenzene, MEK: Methyl Ethyl Ketone, Sty: Styrene, Tol: Toluene, m,p-X: m,p-Xylene, o-X: o-Xylene, p-DCB: p-Dichlorobenzene.

The weekday vs. weekend difference was examined on the selected 10 VOCs. We found significant higher concentrations of dichloromethane, MEK and toluene on weekdays than those on weekends (p<0.05). These VOCs are commonly used in industrial products. For example, large quantity of MEK and toluene was emitted to atmosphere from industrial facilities located in Paterson area according to the NJDEP’s EI database. In addition, dichloromethane is widely used as an industrial solvent/degreaser, paint stripper, aerosols and pesticides [30]. The ratios of weekday/weekend averages of the 10 VOC species at each sampling site are plotted in Figure 3. The bar charts above the dotted line (ratio of 1.0) mean that the average weekday concentration was higher than the weekend concentration. Most VOCs were measured higher on weekdays than on weekends at the three monitoring sites in Paterson, indicating greater industrial and commercial activities on weekdays. Elevated VOCs on weekdays suggested the impact of emissions from industrial facilities and commercial districts on VOC air pollution in Paterson. The analysis of weekday vs. weekend ratio confirmed the findings from the spatial analysis, i.e., significant impact was found from the emissions generated by traffic, commercial activities and the operation of industrial facilities located in Paterson.

### Source Apportionment

**Figure 4:**
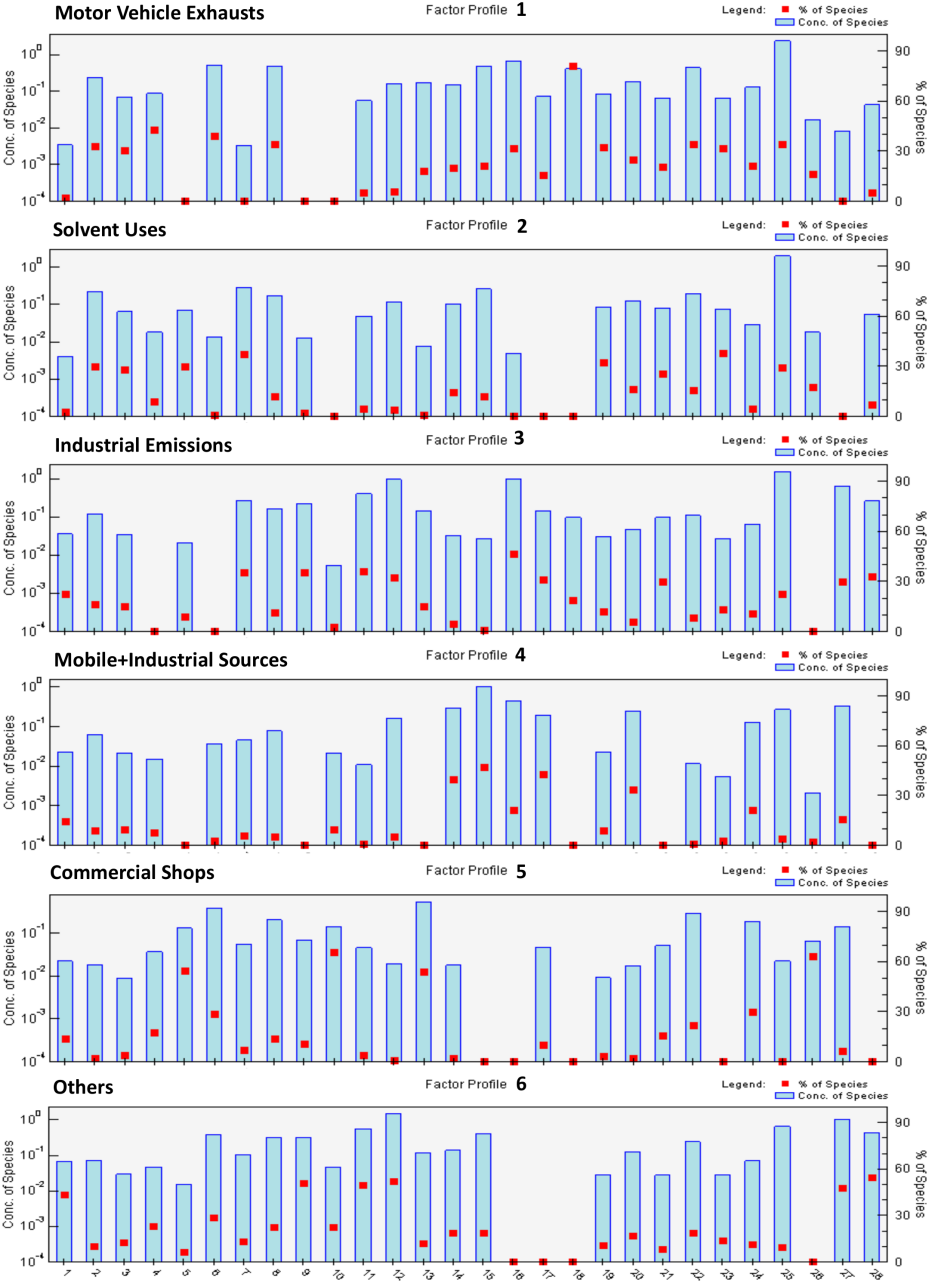
Factor profiles for ambient VOC data collected in 2005∼2006 in Paterson, NJ. Abbreviation in the figure: 1: 1,1,1-Trichloroethane, 2: 1,2,4-Trimethylbenzene, 3: 1,3,5-Trimethylbenzene, 4: 1,3-Butadiene, 5: Acetonitrile, 6: Acetylene, 7: Acrolein, 8: Benzene, 9: Carbon Tetrachloride, 10: Chloroform, 11: Chloromethane, 12: Dichlorodifluoromethane, 13: Dichloromethane, 14: Ethylbenzene, 15: m,p-Xylene, 16: Methyl Ethyl Ketone, 17: Methyl Isobutyl Ketone, 18: Methyl tert-Butyl Ether, 19: n-Octane, 20: o-Xylene, 21: p-Dichlorobenzene, 22: Propylene, 23: Styrene, 24: Tetrachloroethylene, 25: Toluene, 26: Trichloroethylene, 27: Trichlorofluoromethane, 28: Trichlorotrifluoromethane.

**Figure 5:**
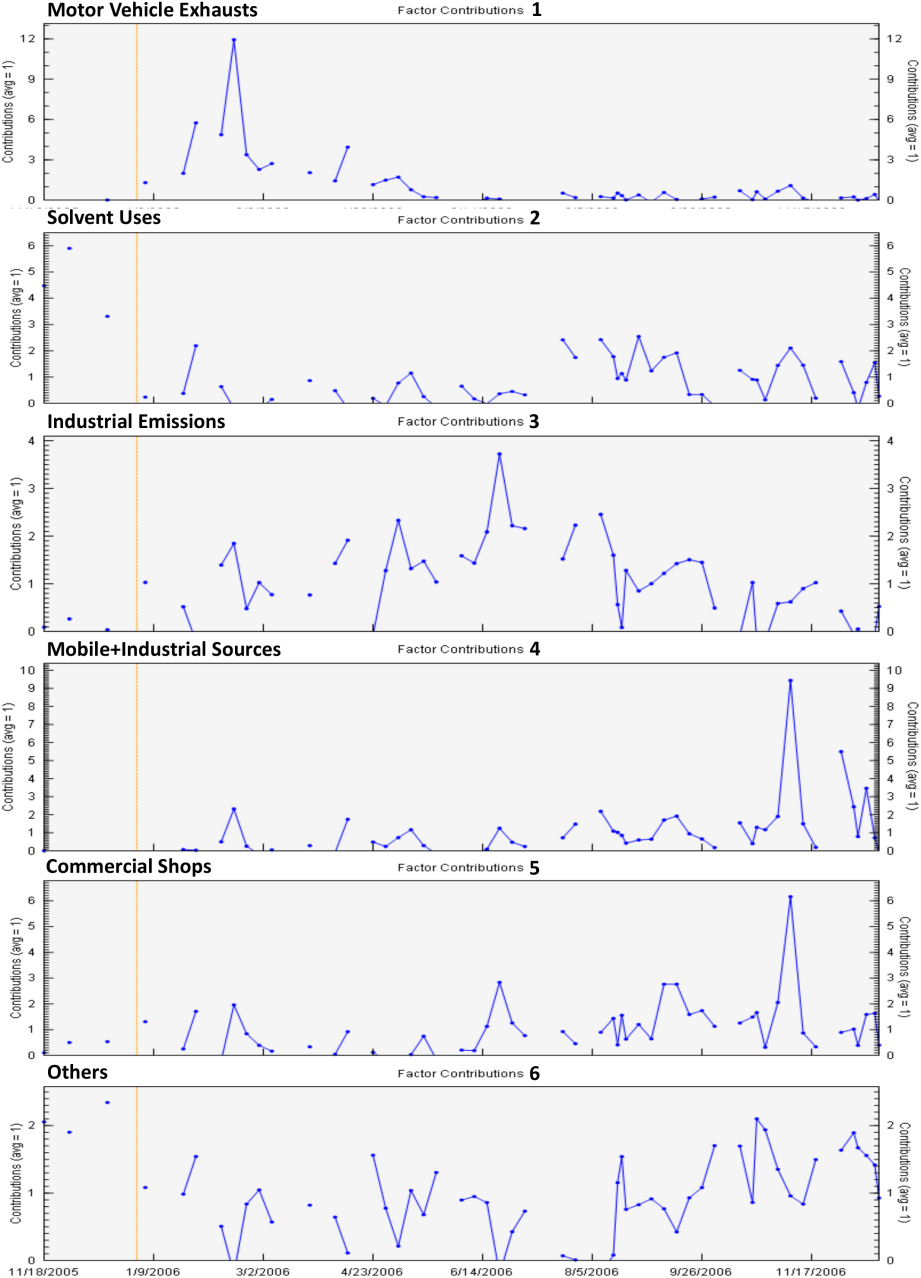
Factor contributions for the duration of the study (11/18/2005∼12/19/2006) in Paterson, NJ.

**Figure 6:**
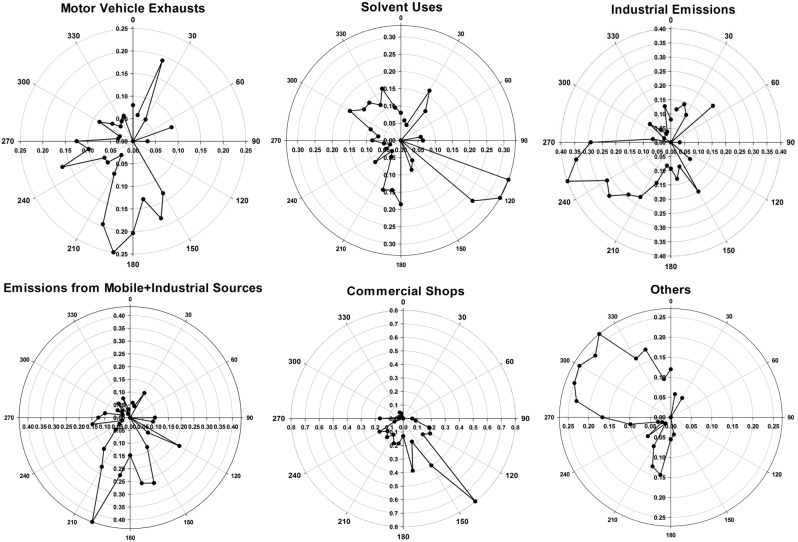
Hourly CPF plots for the highest 10% of the mass contribution from VOC sources in Paterson, NJ.

**Figure 7:**
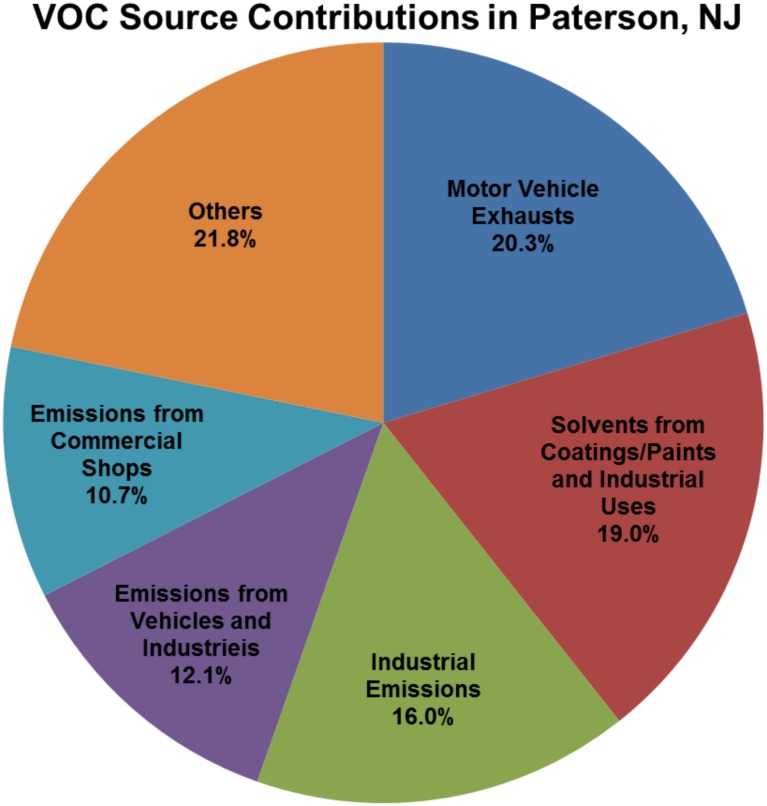
The VOC source contributions to ambient air in Paterson, NJ.

We identified 6 VOC source profiles from our community-scale ambient VOC monitoring data. The resolved factor profiles and the source contributions are presented in Figures 4 and 5, respectively. In Figure 4, each source profile was displayed by a log-scaled mass concentration (µg/m^3^) on the primary y-axis and %species on the secondary y-axis, respectively. The left axis represents a mass concentration apportioned by each species, and the right axis indicates the contribution of each species to the source profile. In Figure 5, the source contributions indicate temporal changes in contribution influenced by meteorological factors and emission intensities. Hourly CPF for the highest 10% of the VOC mass contribution is plotted with wind direction data in Figure 6. Higher calculated CPF represents stronger impact from the point source in a given time and direction, suggesting potential locations for the PMF-identified sources. The overall contribution of each factor to total VOC mass is provided in Figure 7. The quality of the PMF solutions was evaluated by comparing the reconstructed VOC mass contributions (the sum of the contributions from the PMF resolved factors) with the measured VOC mass concentrations. The results showed a good agreement (slope = 0.94 and r^2^ = 0.95) between the two VOC concentrations, indicating that the resolved factors well reproduced measured values and accounted for most of the variations in the measured VOC mass concentrations.

Based on the source profiles, the temporal patterns, i.e., higher VOCs concentrations in the winter and/or higher on weekdays, and the source information from the NJDEP’s EI database, we identified possible sources for each factor profile. The most significant factor in Paterson was motor vehicle exhausts (20%), driven by MTBE, acetylene and benzene in the winter. Similar source profiles for ambient VOCs were also reported in previous studies [13], [14]. The dominant source direction was south, suggested by the hourly CPF plot in Figure 6. Heavy traffics were concentrated on the highways located in the south of Paterson. The second most significant factor was solvent emissions from coatings/paints and industrial uses (19%). This source profile was characterized by higher contributions of toluene, styrene and n-octane in the fall and on weekdays. These results are consistent with the source emission data. As well documented, toluene is widely used for solvents in coatings/paints and industrial processes [30]. Annual emissions of toluene (10.5 tons) and styrene (0.45 ton) were reported to the NJDEP for the year of 2006 in Paterson. The hourly CPF plot also indicated the source direction primarily from the northwest and southeast. The heavily industrialized area known as “Bunker Hill” was located in the north of Paterson city. In addition, many other industrial facilities were clustered in the south or southeast of Paterson. The third significant factor in Paterson was industrial emissions (16%), specifically dominated by MEK and MIBK in the source profile. According to the NJDEP’s EI database, annual MEK and MIBK emissions were 9.1 tons and 3.2 tons in the air from the industrial facilities located in Paterson. The hourly CPF plot indicated that those MEK and MIBK emissions were dominant by the industrial facilities located in the southwest and northeast of Paterson. A hot stamping/metal foil manufacturing facility was located in the south of Paterson city, emitting 5.8 tons of MEK and 3.2 tons of MIBK per year. A coating and laminating facility was located in the north of Paterson, emitting MEK with a rate of 3.3 tons/year. The fourth contributing factor was the combined emissions from industrial and vehicle sources (12%), represented by stronger contributions from ethylbenzene, m,p-xylene and o-xylene in the profile. These results are consistent with the source emission data. According to the NJDEP’s EI database in 2006, 8.4 tons xylenes and 1.6 tons ethylbenzene were emitted to the air annually in Paterson. Motor vehicle emissions also contain significant amount of these species [30]. The hourly CPF plot showed that the sources were significantly originated from the south of Paterson, where heavy trafficked highways and industrial facilities were located. A chemical plant was located in the southeast of Paterson, emitting significant amounts of xylenes and ethylbenzene (annually 8.2 tons and 1.6 tons, respectively) to ambient air in Paterson. The fifth contributing factor was emissions from commercial activities in downtown of Paterson. Included were dry cleaners, nail salons and printing press (11%). This source profile was apparently contributed by trichloroethylene, tetrachloroethylene and dichloromethane in heating seasons and on weekdays. Trichloroethylene and tetrachloroethylene are solvents widely used for degreasing in dry cleaning [30]. The hourly CPF plot supports the VOC emissions related to commercial activities in downtown of Paterson substantially located in the south of Paterson. The sixth contributing factor was noticeable by higher contributions from carbon tetrachloride and chloromethane seen in Figure 4 as well as relatively constant contribution during entire study period observed in Figure 5. Carbon tetrachloride (0.62±0.17 µg/m^3^) and chloromethane (1.15±0.20 µg/m^3^) in Paterson were similar to those at the background site in Chester (0.58±0.18 and 1.14±0.18 µg/m^3^, respectively). They are very stable in the atmosphere and there are no significant local emission sources [30]. In particular, carbon tetrachloride has been phased out in consumer products since 1970. Thus, there are no significant anthropogenic sources for these two species in Paterson. We consider this factor profile indicated aged (background) VOCs in the air and collectively named as “Others” in the pie chart (22%).

Assuming each source contributes equally to the mixed source profiles (i.e., factor profiles 2 and 4), we can classify those contributing sources into three broad categories: industrial (32%), mobile (26%) and commercial (20%) in ambient air of Paterson. The remaining 22% represents aged VOCs in the atmosphere or un-identified potential VOC sources (e.g., evaporative emissions and liquid gasoline) in urban air. The results from the source identification showed that the impact from the land use type, i.e., commercial, industrial and mobile sources, on Paterson VOC air pollution is similar. Thus, we would not expect to see significant spatial differences for many VOCs in Paterson, as observed in the study (see the section of Spatial Variability). This study was the first effort to conduct source apportionment for the one year measurements of relatively stable VOCs in ambient air. The study demonstrated that the 24-hour averaged VOC data were successfully used for the PMF source apportionment. This is because the sources identified in the study were consistent with those from previous source apportionment studies in Northeast areas [15], [35], [36]. They were also consistent with the results derived from the PM_10_ [17] and PAHs [16] measured concurrently. Lin et al. [16] indicated that diesel emissions, combustion of oil, coal and fossil fuels were dominant PAH sources in Paterson. Yu et al. [17] reported seven contributing sources to ambient PM_10_ in downtown of Paterson: sulfate aerosols (26%), vehicle emissions (16%), residual oil combustions (16%), industrial emissions (12%), airborne soils (12%), road dusts (13%) and road salts (5%).

### Limitations and Recommendation

There are some limitations of the study. First, the PMF model could not identify all of the contributing sources to local VOCs because some signature VOCs (e.g., ethane, pentane, propane, isoprene, etc.) of potential sources, such as evaporated gasoline vapor, liquid gasoline, natural gas and biogenic emissions, were not measured in the study. Second, VOC sampling in Paterson were conducted on the roof-top of the 2–4 story buildings, which may tend to underestimate the ground-level VOC emissions including traffic sources. However, the VOC concentrations measured on the rooftop (i.e., 10∼13 meters above the ground) were not significantly lower than the measurements on the ground-level (approximately 2%). Thus, the underestimated VOC concentrations do not significantly affect the conclusions drawn in the study. A risk assessment study is recommended for further evaluation of potential health risks based on the measurements of the community-scale monitoring study.

## Conclusions

This study monitored 60 VOCs in Paterson, NJ, an urban community with mixed sources of air pollution, and characterized spatial/temporal variations of 10 VOCs that were detected over 75% during the course of monitoring period, toxic and/or having known sources in the study area. The study demonstrated that monitoring VOCs at community scale was an effective approach to capture spatial/temporal variations of VOCs in an urban area with mixed VOC sources. The comparisons between sites and between weekday and weekend indicated the significant impact from anthropogenic VOC emissions on ambient air pollution in Paterson. These observations are consistent with the NJDEP EI database. The PMF source apportionment results confirmed the contributions from various sources of VOCs in Paterson, including industrial (32%), mobile (26%) and commercial (20%) emission sources. The estimated source contributions from our study are more accurate than those obtained from the summer measurements only. The findings from this study demonstrated the importance of the community-oriented air toxic monitoring approach because it was able to 1) capture spatial variation in VOCs, 2) identify sources of concern (contribution from different emission sources), 3) better assess community exposure to ambient VOC air pollution, and 4) develop effective controlling strategies for urban communities with mixed air pollution sources.
